# Generative Artificial Intelligence Self-Efficacy and Learning Engagement Among Special Education Teacher Trainees: A Moderated Mediation Model

**DOI:** 10.3390/bs16040488

**Published:** 2026-03-26

**Authors:** Xiage Liu, Juan Yang, Wei Zhao, Tingzhao Wang

**Affiliations:** 1 Faculty of Education, Shaanxi Normal University, Xi’an 710062, China; jy20240906@snnu.edu.cn (J.Y.); wangtingzhao@snnu.edu.cn (T.W.); 2 Normal College, Xianyang Polytechnic Institute, Xianyang 712000, China

**Keywords:** GenAI, self-efficacy, learning engagement, critical thinking, problem-solving ability

## Abstract

Generative artificial intelligence (GenAI), known for its personalization and intelligence, is gaining traction in special education as a tool to address diverse learner needs. As future key practitioners integrating intelligent technology to promote educational equity, how special education teacher trainees effectively utilize GenAI has become a critical issue. In the context of Chinese higher education, this study employed a cross-sectional design and administered a questionnaire survey to 434 special education teacher trainees. The aim was to examine the association between their GenAI self-efficacy and learning engagement, with particular attention to the potential mediating association of problem-solving ability and the moderating role of critical thinking. The results revealed the following: GenAI self-efficacy was positively associated with learning engagement, and problem-solving ability played a mediating role in the relationship between self-efficacy and learning engagement. Moreover, critical thinking significantly moderated the relationship between self-efficacy and problem-solving ability.

## 1. Introduction

Generative artificial intelligence (GenAI) is reshaping the special education ecosystem, emerging as a core technology for promoting educational equity and quality (Fan et al., 2025). As information technology in special education advances from a digital foundation towards digital-intelligent integration (M. Deng et al., 2022), GenAI operates through the synergy of large-scale network data and large language model algorithms. It possesses the ability to process multimodal information and generate dynamic content, enabling personalized adaptation for the highly heterogeneous population in special education (e.g., students with visual impairments, hearing impairments, autism spectrum disorder, etc.) (L. Deng & Lei, 2021; Wei & Li, 2025). This provides a new technological pathway to transcend the limitations of traditional education and achieve enhanced quality and efficiency.

However, this wave of intelligent advancement also creates a structural challenge: a widening gap between the rapid evolution of intelligent technologies and special education teacher trainees’ capacity to apply them effectively (Kong, 2023). This mismatch suggests that the primary bottleneck in technology-enhanced education lies less in the technology itself than in human capability. Consequently, the professional roles and competency frameworks of special education teachers are being redefined, with the effective integration of GenAI shifting from a supplementary skill to a core professional requirement. Rather than merely applying generic models, GenAI use in special education requires inclusive and adaptive design tailored to students with diverse disabilities (Confer, 2023). Although GenAI can support scaffolded instruction and personalized learning assistance, its effectiveness ultimately depends on teachers’ ability to embed it meaningfully within instruction and adjust practices based on student feedback (Fan et al., 2025). Thus, future special education teachers must develop not only technical knowledge but also the capacity for flexible and critical integration of GenAI into differentiated teaching to address students’ highly individualized learning needs.

The realization of technological potential in special education is fundamentally contingent upon teachers’ professional competence, particularly that of teacher trainees as a critical reserve of the future workforce. However, emerging technologies in education exhibit a dual nature, simultaneously offering empowerment and risks. Owing to data and algorithmic limitations, GenAI may produce “technological hallucinations,” generating inaccurate outputs that undermine learning effectiveness. Over-reliance on these tools may further trigger academic integrity concerns and inhibit the development of higher-order cognitive skills (Su, 2025). Meanwhile, students’ technology use is often characterized by a cognition–practice gap, in which positive attitudes fail to translate into effective application, resulting in relatively low levels of GenAI-related self-efficacy. Self-efficacy has been identified as a key predictor of learning engagement and persistence and demonstrates strong explanatory power in intelligent learning environments (M. K. Zhang et al., 2021; Wan et al., 2021). For teacher trainees, technology-related self-efficacy directly influences their willingness to participate in and sustain technology-integrated instructional practices (Kong, 2023). Against the backdrop of rapid AI advancement, enhancing special education teacher trainees’ competence and confidence in using GenAI is not only a practical necessity for addressing the complex demands of special education but also an essential prerequisite for promoting educational equity.

### 1.1. GenAI Self-Efficacy and Academic Engagement

Grounded in social cognitive theory, self-efficacy denotes individuals’ context-specific beliefs about their capability to organize and execute actions required for task accomplishment (Bandura, 1997; Bandura, 2006). Extending this perspective, GenAI self-efficacy is conceptualized as perceived competence in effectively leveraging GenAI systems to support learning and teaching within human–AI collaboration. In contrast to general academic self-efficacy, which reflects broad academic confidence, GenAI self-efficacy captures technology-situated competencies, including a critical evaluation of AI-generated outputs, strategic interaction skills (e.g., prompt design and iterative refinement), and adaptive regulation within dynamic technological environments. This construct is operationalized through the four-dimensional framework proposed by Y. Y. Wang and Chuang (2024): perceived utility, anthropomorphic interaction, comfort, and technical proficiency.

GenAI-supported learning engagement, in turn, refers to learners’ sustained and active involvement in task completion within human–AI collaborative contexts. Drawing on the tripartite framework of behavioral, cognitive, and affective engagement (Fredricks et al., 2004), it encompasses strategic tool use and interaction, higher-order processing of AI-generated content, and emotional experiences associated with technology use.

Despite the pedagogical affordances of intelligent technologies, the overall integration of AI into learning remains limited. University students’ use of intelligent tools is frequently superficial, characterized by direct adoption and task completion rather than a systematic application of higher-order cognitive strategies (Li et al., 2025). Consequently, AI-assisted learning is often confined to low-level functions such as information retrieval and assignment support, constraining meaningful and sustained engagement.

This limitation is particularly evident among teacher trainees. Although they acknowledge the transformative potential of AI, their practices tend to focus on basic operations rather than instructional redesign or pedagogical innovation. Empirical evidence further indicates divergent perceptions of AI—ranging from threat to opportunity—yet systematic competence support remains insufficient (Yuan et al., 2025). As a result, their learning engagement is often fragmented and surface-level, falling short of the deep and structured participation required to fully realize the affordances of GenAI.

Against this backdrop, GenAI self-efficacy emerges as a critical psychological mechanism underlying learning engagement. Learners’ confidence in their capability to effectively utilize GenAI fosters stronger expectancy–value beliefs, goal-directed behaviors, and positive affective reinforcement, thereby promoting sustained involvement. Students with higher self-efficacy are more likely to adopt deep learning strategies, including critical analysis of AI-generated content, interdisciplinary knowledge integration, and transfer-based application (X. Zhou & Lou, 2021). Such higher-order cognitive processing and proactive behaviors directly facilitate greater cognitive and behavioral engagement, suggesting that strengthening GenAI self-efficacy may represent a key pathway for enhancing meaningful learning participation among teacher trainees.

### 1.2. The Mediating Role of Problem-Solving Ability

Both problem-solving and critical thinking represent essential higher-order cognitive competencies in the digital age (Pellas, 2025). Problem-solving is generally conceptualized as an iterative and collaborative process involving the coordination of strategies, integration of diverse knowledge, and construction of shared cognitive frameworks to address complex tasks (Larson & Christensen, 1993). Accordingly, problem-solving ability denotes the capacity to apply systematic cognitive strategies—such as analysis, reasoning, and reflection—to identify problems, integrate resources, and generate effective solutions in unstructured and cross-domain contexts.

Within GenAI-supported environments, these processes can be substantially enhanced. By leveraging GenAI’s capabilities in language comprehension, knowledge synthesis, and technical simplification, learners can compensate for cognitive constraints, including shallow reasoning, inefficient decision-making, and limited reflective transfer (Moșoi et al., 2025). In this regard, GenAI self-efficacy serves as a critical psychological driver of technology-assisted problem-solving. Higher self-efficacy strengthens learners’ cognitive confidence and perceived control, increasing their willingness to engage GenAI as a cognitive partner for task decomposition, resource expansion, and solution generation. Empirical evidence from STEM education further demonstrates that enhanced self-efficacy significantly improves teachers’ creative problem-solving performance (Durnali & Gökbulut, 2025).

Problem-solving, in turn, functions as an important pathway to deeper learning engagement. As an inherently metacognitive and cognitively demanding process, problem-solving requires sustained attention, strategic regulation, and active knowledge construction, all of which reflect substantial cognitive investment. Studies indicate that metacognitive monitoring—a core component of problem-solving—positively predicts the depth and persistence of learning engagement (Sheng, 2024). Moreover, the sense of competence and satisfaction derived from successful technology use further reinforces engagement. Consistent with constructivist perspectives, problem-solving and critical thinking have been identified as key mechanisms linking learning processes to higher engagement and academic achievement (Almulla, 2023).

### 1.3. The Moderating Role of Critical Thinking

Grounded in the interactive principle of person–behavior–environment in social cognitive theory, this study conceptualizes critical thinking as a key individual cognitive trait that serves as a moderating variable. Specifically, we focus on its moderating role in the first segment of the mediating pathway (i.e., self-efficacy → problem-solving ability), as this stage represents the crucial process through which internal beliefs are transformed into external cognitive actions.

Critical thinking functions as both a strategic optimizer and a risk mitigator in the relationship between self-efficacy and problem-solving. Learners with high levels of critical thinking are not satisfied with merely utilizing the basic functions of GenAI. Instead, driven by strong self-efficacy, they actively apply analytical, inferential, and evaluative skills to design more sophisticated prompt chains, conduct multiple rounds of verification and iteration, and ultimately develop deeper and more systematic problem-solving strategies (Chu et al., 2025).

Moreover, when confronted with potential hallucinations in GenAI outputs (Sun et al., 2024), critical thinking serves as a cognitive filter. It prevents learners with high self-efficacy from blindly trusting technological outputs and encourages them to maintain prudent skepticism, verify information, trace sources, and ensure that their problem-solving processes are grounded in reliable evidence (Mariyono & Alif Hidayatullah, 2025). This “active yet prudent” human–AI interaction mode is essential for effectively converting high self-efficacy into high capability.

Consequently, at the same level of self-efficacy, students with stronger critical thinking are better able to translate technological confidence into high-quality analysis, reasoning, and generative behaviors, thereby demonstrating superior problem-solving ability. In summary, critical thinking is expected to exert a positive moderating effect on this relationship: as critical thinking increases, the positive impact of GenAI self-efficacy on problem-solving ability becomes stronger.

Although artificial intelligence provides teacher education students with abundant instructional resources and efficient learning tools, it also introduces new challenges regarding the wise use of technology and engagement in deeper thinking (Y. Zhang et al., 2025). Teacher trainees face dual identity demands: as learners, they must use intelligent technologies to support applied learning; as future teachers, they must wisely employ these technologies to facilitate others’ learning. Promoting the transition from merely using to wisely employing technological tools through critical thinking is therefore an essential requirement in their professional development within human–AI collaborative environments.

Based on the above reasoning, this study proposes the following hypotheses:

**H1.**  *GenAI self-efficacy among special education teacher trainees positively predicts their learning engagement.*

**H2.**  *Problem-solving ability in the context of GenAI mediates the relationship between self-efficacy and learning engagement. Specifically, GenAI self-efficacy was positively associated with problem-solving ability, and problem-solving ability was positively associated with learning engagement. Furthermore, GenAI self-efficacy was indirectly associated with learning engagement through problem-solving ability.*

**H3.**  *Critical thinking positively moderates the first stage of the mediating pathway (self-efficacy → problem-solving ability), such that higher levels of critical thinking strengthen the positive effect of GenAI self-efficacy on problem-solving ability.*

## 2. Materials and Methods

### 2.1. Research Procedures and Participants

This study adopted a convenience sampling method and conducted a cross-sectional survey via an online questionnaire platform targeting undergraduate students majoring in special education at universities in four provinces: Shaanxi, Sichuan, Yunnan, and Hainan.

Participant screening followed explicit inclusion and exclusion criteria. The inclusion criteria were (1) full-time undergraduate students currently enrolled in a special education program; (2) possessing basic knowledge of or experience with the application of GenAI in education; and (3) voluntary participation with selection of the “Agree” option after fully reading the informed consent statement on the questionnaire’s first page. The exclusion criteria were (1) not meeting the specified major or educational level requirements; (2) questionnaire completion time being less than 50% of the average completion time or exhibiting patterned responding; and (3) failure to complete the informed consent process or to submit a fully answered questionnaire. A total of 503 questionnaires were collected. Following the aforementioned screening procedures, 434 valid responses were obtained, representing a response rate of 86.29%.

### 2.2. Assessment

#### 2.2.1. GenAI Self-Efficacy Scale

This study employed the AI Self-Efficacy Scale developed by Wang Yingyao and Chuang Yenwen (Y. Y. Wang & Chuang, 2024) to measure individuals’ generalized belief in their ability to use and interact with artificial intelligence. A comprehensive localization process, including translation, expert review, and pilot testing, was conducted for the generative AI self-efficacy measurement tool to ensure its applicability. The scale consists of 22 items across four dimensions: Assistance (7 items), Human–Computer Interaction (5 items), Comfort with AI (6 items), and Technical Skills (4 items). Responses were recorded on a 7-point Likert scale ranging from 1 (“strongly disagree”) to 7 (“strongly agree”), with higher scores indicating greater AI self-efficacy.

To examine the structural validity of the scale within our sample, a confirmatory factor analysis (CFA) was conducted. The results showed that χ^2^/df = 2.69, RMSEA = 0.06, and SRMR = 0.06, indicating acceptable model fit based on these absolute fit indices. However, the incremental fit indices, TLI (=0.86) and CFI (=0.85), were slightly below the 0.90 threshold for excellent fit, falling within the acceptable range. To evaluate the scale’s reliability and validity more comprehensively, additional evidence was examined: the composite reliability (CR) for each dimension ranged from 0.75 to 0.81, all exceeding the 0.70 benchmark. Furthermore, the standardized factor loadings for all items ranged from 0.78 to 0.82 (all > 0.50) and were statistically significant. Additionally, the scale demonstrated excellent overall internal consistency (Cronbach’s α = 0.92).

#### 2.2.2. GenAI Problem-Solving Ability

The measurement of problem-solving ability referenced the scale employed by Zhou Xue, Teng Da, and Hosam Al-Samarraie (X. Zhou et al., 2024). This scale originated from the problem-solving items in the self-control behavior scale developed by Rosenbaum (Rosenbaum, 1980). It consists of 11 items forming a unidimensional structure, rated on a 7-point Likert scale ranging from “strongly disagree” (1) to “strongly agree” (7). Higher scores indicate a higher level of problem-solving ability. Confirmatory factor analysis showed the following fit indices: χ^2^/df = 2.05 (less than 3, indicating good model fit), RMSEA = 0.04, SRMR = 0.04, TLI = 0.96, and CFI = 0.97, all within acceptable ranges. The Cronbach’s α coefficient for this scale was 0.91.

#### 2.2.3. GAI Critical Thinking

The measurement of critical thinking referenced the scale employed by Zhou Xue, Teng Da, and Hosam Al-Samarraie (X. Zhou et al., 2024). This scale originated from Pintrich’s Motivated Strategies for Learning Questionnaire (MSLQ) (Pintrich, 1991). The final scale used in this study has a unidimensional structure consisting of 5 items, rated on a 7-point Likert scale ranging from “strongly disagree” (1) to “strongly agree” (7). In the formal sample of this research, confirmatory factor analysis confirmed its good structural validity: χ^2^/df = 1.86 (below 3, indicating good model fit), RMSEA = 0.07, SRMR = 0.02, TLI = 0.98, and CFI = 0.96, all within acceptable ranges. The Cronbach’s α coefficient for this scale was 0.85.

#### 2.2.4. Learning Engagement

Learning engagement was measured using the Chinese version of the scale originally developed by Schaufeli, which was translated by Fang Laitan (Fang et al., 2008). The scale consists of 17 items divided into three dimensions: Vigor (6 items), Dedication (5 items), and Absorption (6 items). Items were rated on a 7-point Likert scale from “strongly disagree” (1) to “strongly agree” (7), with higher scores indicating a higher level of learning engagement. The model fit indices showed χ^2^/df = 1.83, meeting the excellent criterion of being below 3; SRMR = 0.04, TLI = 0.91, and CFI = 0.92, all reaching the recommended level of >0.90, indicating excellent relative fit and residual control. However, the RMSEA value was 0.09, slightly above the stringent threshold of 0.08 but still within the acceptable range of <0.10. Considering all indices comprehensively, particularly the excellent values of χ^2^/df, TLI, CFI, and SRMR, it can be concluded that the three-factor model demonstrates an overall acceptable fit to the data. Furthermore, the scale exhibited extremely high internal consistency in this study (Cronbach’s α = 0.96).

### 2.3. Data Analysis

Descriptive statistics, correlation analyses, and tests for common method bias were conducted using SPSS 26.0. Moderated mediation effects were examined using the PROCESS macro (Version 4.0). Gender and major were included as covariates in the model.

Indirect effects were tested using bias-corrected bootstrapping with 5000 resamples. Statistical significance was determined based on 95% bootstrap confidence intervals (CIs), with effects considered significant when the interval did not include zero.

## 3. Results

### 3.1. Descriptive Statistics and Correlation Analysis of Primary Research Variables

Descriptive statistics, including mean, standard deviation, and correlation analysis, were conducted on special education teacher trainees’ GenAI self-efficacy, problem-solving ability, critical thinking, and learning engagement. Results are presented in Table 1. Self-efficacy exhibited a positive correlation with learning engagement (r = 0.35, *p* < 0.01), problem-solving ability (r = 0.65, *p* < 0.01), and critical thinking (r = 0.54, *p* < 0.01). Critical thinking and problem-solving ability showed a positive correlation (r = 0.63, *p* < 0.01). Subsequently, Pearson correlation analysis was employed to examine the relationships among the variables. The results are presented in Table 1.

### 3.2. Common Method Bias Test

To comprehensively address common method bias, this study implemented procedural preventive measures during the data collection stage and conducted a combined assessment using statistical tests. To mitigate common method bias, procedural measures—including an emphasis on anonymity, authenticity, and confidentiality—were implemented through instructions prior to the assessment.

The Harman single-factor test was employed to examine common method bias (H. Zhou & Long, 2004). Results indicated that 18 factors with eigenvalues exceeding 1 were extracted without rotation. The first factor explained 31.58% of the variance (<40%). Consequently, common method bias appears to have exerted a negligible influence on the findings of this study.

### 3.3. The Mediating Role of Problem-Solving Ability

Mediation effects were tested using Model 4 of the PROCESS macro with GenAI self-efficacy as the independent variable, learning engagement as the dependent variable, and problem-solving ability as the mediator. Indirect effects were estimated using bias-corrected bootstrapping with 5000 resamples. Results are summarized in Table 2.

GenAI self-efficacy significantly and positively predicted learning engagement, with the 95% confidence interval excluding zero (CI = [0.05, 0.33]). The indirect effect through problem-solving ability was also significant (effect = 0.24, CI = [0.11, 0.38]). Consistent with mediation theory (Wen & Ye, 2014), the indirect pathway accounted for 55.8% of the total effect, indicating partial mediation.

### 3.4. The Moderating Role of Critical Thinking

To investigate how critical thinking moderates the first stage of the mediating pathway, this study employed Model 7 from the PROCESS macro to analyze the relationships among variables while controlling for covariates such as gender and grade. All predictor variables were mean-centered prior to analysis. The detailed results are presented in Table 3.

Moderated mediation was tested using Model 7 of the PROCESS macro (Hayes, 2018) to examine whether critical thinking moderated the indirect effect of GenAI self-efficacy on learning engagement through problem-solving ability. All continuous predictors were mean-centered prior to computing interaction terms. Gender and grade were included as covariates but were not statistically significant. The index of moderated mediation was significant (index = 0.03, BootSE = 0.01, 95% CI [0.00, 0.04]), indicating that the indirect effect varied across levels of critical thinking.

Conditional indirect effects were further estimated at the 16th and 84th percentiles of critical thinking (see Table 4). The indirect effect was stronger at higher levels of critical thinking (b = 0.91, 95% CI [0.78, 1.03]) than at lower levels (b = 0.76, 95% CI [0.62, 0.89]). All confidence intervals excluded zero, suggesting that the mediating pathway was amplified as critical thinking increased.

**Figure 1:**
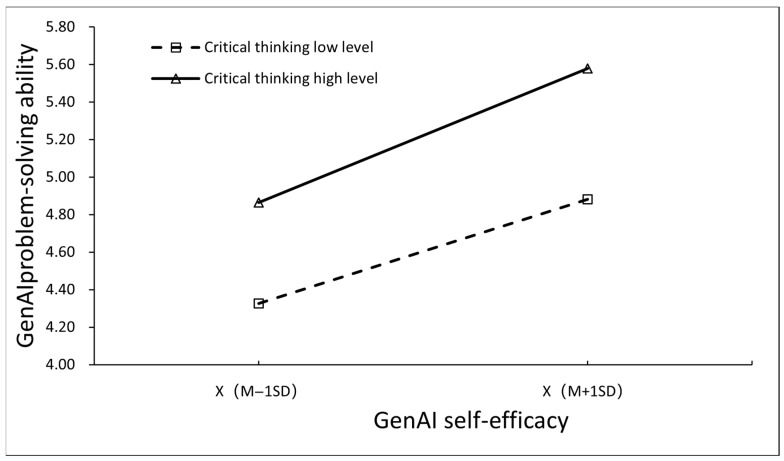
Moderation plot.

As shown in Figure 1, the two lines in the graph are not parallel. The moderating role of critical thinking was significant; when critical thinking was at a high level, the increase in problem-solving ability resulting from an increase in self-efficacy was about 1.19 times larger than that at a low level.

## 4. Discussion

### 4.1. The Impact of GenAI Self-Efficacy on Learning Engagement

The findings reveal a significant positive association between GenAI self-efficacy and learning engagement among special education teacher trainees, supporting Hypothesis 1. This result extends the applicability of self-efficacy theory to AI-enhanced educational contexts and provides empirical evidence for the digital transformation of special education teacher preparation. Given the complexity of integrating intelligent technologies into inclusive teaching, trainees’ psychological readiness—particularly their confidence in using GenAI—appears to be a critical enabling factor.

This interpretation is consistent with prior research. Studies in special education have shown that teachers’ perceptions and acceptance of assistive technologies are closely associated with their classroom implementation and effectiveness (Voultsiou & Moussiades, 2025), while research in online learning environments similarly has identified a positive relationship between digital self-efficacy and learning engagement (B. Liu et al., 2023).

From a self-regulation perspective, GenAI self-efficacy may facilitate engagement through multiple, interrelated pathways. Higher self-efficacy is associated with greater behavioral exploration of advanced GenAI functions, enhances cognitive regulation through more effective information screening and integration, and strengthens affective experiences by reducing technology-related anxiety and fostering positive human–AI interaction (C. Chen et al., 2024; Y. Liu et al., 2025; Lu et al., 2022). Together, these behavioral, cognitive, and affective processes may interact in ways that are conducive to sustaining and deepening learning engagement.

### 4.2. Mediating Role of Problem-Solving Competence

The findings further demonstrate that problem-solving ability mediates the relationship between GenAI self-efficacy and learning engagement among special education teacher trainees, supporting Hypothesis 2. In addition to its direct association with engagement, GenAI self-efficacy indirectly promotes engagement by enhancing learners’ problem-solving competence. This mediation pathway—self-efficacy → problem-solving ability → learning engagement—extends current understandings of learning mechanisms in AI-supported environments and highlights that the value of technological self-efficacy operates partly through higher-order cognitive processes.

This interpretation aligns with prior evidence. A systematic review of GenAI applications in higher education indicates that students’ beliefs about the utility of GenAI and their perceived capability to use it are closely associated with active learning behaviors, while GenAI environments are also linked to the development of problem-solving skills (Wu et al., 2025). Learners can progressively strengthen such skills through iterative cycles of exploration, solution generation, and reflection when interacting with intelligent systems (Santiago, 2025).

GenAI-supported problem-solving is associated with higher levels of engagement across multiple dimensions. Cognitively, AI-assisted information filtering, integration, and strategy generation are related to deep processing and metacognitive regulation. Behaviorally, the automation of routine tasks is associated with shifts in cognitive resources toward solution implementation and innovation, as evidenced by improved strategy use and sustained self-directed practice among pre-service teachers trained with GenAI simulations (Lim et al., 2025). Effectively, successful problem resolution and real-time feedback are positively related to learners’ autonomy, interest, and persistence (C. Chen et al., 2024; X. Wang et al., 2025). Together, these cognitive, behavioral, and emotional gains create a reinforcing mechanism through which problem-solving competencies are linked to sustained learning engagement.

### 4.3. The Moderating Effect of Critical Thinking

The results further indicate that critical thinking moderates the relationship between GenAI self-efficacy and problem-solving ability, supporting Hypothesis 3. Specifically, critical thinking may act as a regulatory mechanism that shapes how self-efficacy translates into effective technology use. Learners with stronger critical thinking skills tend to engage in reflective and iterative interaction with GenAI—systematically evaluating, verifying, and integrating AI-generated outputs—which is linked to higher-quality self-regulation and more effective problem-solving (X. Zhou et al., 2024; Y. Zhang et al., 2025; Martínez et al., 2025). In contrast, those with weaker critical thinking may convert high self-efficacy into overconfidence and uncritical reliance on AI, prioritizing rapid answer adoption over deep processing. Such superficial regulation can constrain, or even undermine, the development of problem-solving competence, consistent with evidence of a negative association between excessive trust in intelligent technologies and critical thinking (Hou et al., 2025).

This moderating role highlights the potential importance of critical thinking as a metacognitive factor. While GenAI offers powerful cognitive assistance, its educational benefits may be contingent on learners’ capacity to critically interrogate algorithmic outputs rather than passively accept them.

These considerations are particularly salient in special education. GenAI is increasingly reshaping instructional strategies and classroom practices, offering adaptive and personalized support for students with diverse needs, including those with ADHD or autism spectrum disorder (Ceallaigh et al., 2025). When combined with immersive technologies such as extended reality, GenAI can facilitate customizable learning environments that are related to improvements in perceptual, cognitive, and social skill development, thereby advancing inclusive education (Barbu et al., 2025; Corrigan et al., 2023; Y. Chen et al., 2022). Moreover, GenAI can function as an intelligent scaffold, assisting teachers in generating task analyses, multisensory materials, and preliminary Individualized Education Programs, which are associated with reductions in routine workload and may allow for greater focus on professional judgment and emotional support (Goldman et al., 2024).

Given the vulnerability of learners in special education and the high stakes of instructional decisions, however, the integration of GenAI should be considered in the context of rigorous ethical and professional standards. Future teachers, therefore, require not only technological proficiency but also strong critical evaluation skills to identify algorithmic bias, inaccuracies, and hallucinated content, ensuring that AI applications are aligned with equity and serve students’ best interests rather than inadvertently reinforcing existing disparities.

### 4.4. Limitations and Future Directions

The present study contributes to the literature by focusing on special education teacher trainees, a professionally significant yet underrepresented population in research on the educational application of GenAI, thereby enriching the empirical evidence in this emerging field. By integrating self-efficacy, problem-solving ability, and critical thinking, the study develops and tests a moderated mediation model, offering a more nuanced theoretical account of the mechanisms through which GenAI influences learning engagement.

Several limitations warrant consideration. The cross-sectional design and reliance on self-reported measures permit the identification of associations but preclude causal inference. In addition, the use of online convenience sampling may constrain the generalizability of the findings.

Building on these limitations, future research should employ longitudinal or experimental designs to clarify causal relationships, adopt stratified sampling across broader geographic regions and institutional contexts to enhance representativeness, and incorporate multi-method data sources (e.g., behavioral logs and performance assessments) to strengthen measurement validity. Extending the model to in-service teachers and other disciplinary domains would further test its generalizability and boundary conditions.

## 5. Conclusions

The findings demonstrate that GenAI self-efficacy is positively associated with learning engagement among special education teacher trainees, with problem-solving ability serving as a mediating mechanism and critical thinking functioning as a significant moderator of the relationship between self-efficacy and problem-solving. Together, these results highlight the joint roles of motivational beliefs, higher-order cognitive skills, and reflective thinking in shaping technology-supported learning engagement.

From a practical perspective, these findings suggest the need to design instructional tasks that encourage teacher trainees to purposefully utilize diverse GenAI tools to develop inclusive education competencies while simultaneously cultivating critical thinking. Such practices may strengthen both technological proficiency and confidence in AI-supported teaching.

Future research should incorporate multi-source objective data, such as behavioral logs and simulated teaching performance assessments, and adopt longitudinal or experimental designs to better establish causal relationships.

## Figures and Tables

**Table 1:** Correlation for the main variables (n = 434).

	Skewness/Kurtosis	M (SD)	GenAI Self-Efficacy	Problem-Solving Ability	Critical Thinking	Learning Engagement
GenAI self-efficacy	−0.24; 1.73	4.80 (0.76)	1			
problem-solving ability	0.08; −0.16	4.93 (0.75)	0.65 **	1		
critical thinking	−0.35; 1.91	5.01 (0.76)	0.54 **	0.63 **	1	
learning engagement	−0.56; 1.81	4.36 (0.95)	0.35 **	0.40 **	0.27 **	1

** *p* < 0.01.

**Table 2:** Results of the mediation analysis.

	Effect	Boot SE	LLCI	ULCI	Effect Size Proportion
Direct effect	0.19	0.07	0.05	0.33	44.2%
Indirect effect	0.24	0.06	0.11	0.38	55.8%
Total effect	0.43				

**Table 3:** Moderated mediation effects.

	Problem-Solving Ability M				Learning Engagement Y			
	β	SE	t	CI	β	SE	t	CI
GenAI self-efficacy (X)	0.83	0.04	14.31 ***	[0.47, 0.62]	0.22	0.07	2.65 **	[0.08, 0.36]
problem-solving ability (M)	-	-	-	-	0.35	0.07	5.09 ***	[0.21, 0.49]
Critical thinking (W)	−0.10	0.04	−2.73 ***	[−0.18,−0.03]	-	-	-	-
X × W	0.05	0.20	2.67 **	[0.01, 0.10]	-	-	-	-
Gender	0.01	0.10	0.17	[−0.01, 0.20]	0.23	0.14	2.11 *	[0.20, 0.56]
Grade	−0.20	0.33	−0.60	[−0.09, 0.50]	−0.01	0.05	−0.24	[−0.11, 0.09]

* *p* < 0.05, ** *p* < 0.01, *** *p* < 0.001.

**Table 4:** Conditional indirect effects of GenAI self-efficacy on problem-solving ability at different levels of critical thinking.

Critical Thinking (W) Level	Effect	SE	t	95% CI
−1.39 (16th)	0.76	0.06	11.39 ***	[0.62, 0.89]
0.01 (50th)	0.83	0.05	14.03 ***	[0.71, 0.95]
1.41 (84th)	0.91	0.06	14.92 ***	[0.78, 1.03]

*** *p* < 0.001.

## Data Availability

The raw data supporting the conclusions of this article will be made available by the authors upon request.

## References

[B1-behavsci-16-00488] Almulla M. A. (2023). Constructivism learning theory: A paradigm for students’ critical thinking, creativity, and problem solving to affect academic performance in higher education. Cogent Education.

[B2-behavsci-16-00488] Bandura A. (1997). Self-efficacy: The exercise of control.

[B3-behavsci-16-00488] Bandura A. (2006). Social cognition.

[B4-behavsci-16-00488] Barbu M., Iordache D.-D., Petre I., Barbu D.-C., Băjenaru L. (2025). Framework design for reinforcing the potential of XR technologies in transforming inclusive education. Applied Sciences.

[B5-behavsci-16-00488] Ceallaigh T. J. Ó., O’Brien E., Tømte C., Kulaksız T., Connolly C. (2025). Rethinking teacher education in an AI world: Perceptions, readiness and institutional support for generative AI integration. European Journal of Teacher Education.

[B6-behavsci-16-00488] Chen C., Hu W., Wei X. (2024). From anxiety to action: Exploring the impact of artificial intelligence anxiety and artificial intelligence self-efficacy on motivated learning of undergraduate students. Interactive Learning Environments.

[B7-behavsci-16-00488] Chen Y., Zhou Z., Cao M., Liu M., Lin Z., Yang W., Yang X., Dhaidhai D., Xiong P. (2022). Extended reality (XR) and telehealth interventions for children or adolescents with autism spectrum disorder: Systematic review of qualitative and quantitative studies. Neuroscience & Biobehavioral Reviews.

[B8-behavsci-16-00488] Chu H. C., Lu Y. C., Tu Y. F. (2025). How GenAI-supported multi-modal presentations benefit students with different motivation levels: Evidence from digital storytelling performance, critical thinking awareness, and learning attitude. Educational Technology & Society.

[B9-behavsci-16-00488] Confer C. A. (2023). The use of artificial intelligence to create inclusivity in special education classrooms. Journal of Applied Professional Studies.

[B10-behavsci-16-00488] Corrigan N., Păsărelu C.-R., Voinescu A. (2023). Immersive virtual reality for improving cognitive deficits in children with ADHD: A systematic review and meta-analysis. Virtual Reality.

[B11-behavsci-16-00488] Deng L., Lei J. H. (2021). A knowledge mapping analysis of artificial intelligence application in special education. Chinese Journal of Special Education.

[B12-behavsci-16-00488] Deng M., Zhang L., Zhang Y. (2022). The connotation, characteristics, and direction of informatization construction in special education in China under the background of high-quality education development. Chinese Journal of Special Education.

[B13-behavsci-16-00488] Durnali M., Gökbulut B. (2025). Empowering masters of creative problem solvers: The impact of STEM professional development training on teachers’ attitudes, self-efficacy, and problem-solving skills. Journal of Intelligence.

[B14-behavsci-16-00488] Fan W. X., Shi C. Y., Li K. L., Yang J. F. (2025). Empowering the development of students in special education with artificial intelligence: Application logic and practical paths. Modern Distance Education.

[B15-behavsci-16-00488] Fang L., Shi K., Zhang F. (2008). A reliability and validity study of the Chinese version of the utrecht work engagement scale-student. Chinese Journal of Clinical Psychology.

[B16-behavsci-16-00488] Fredricks J. A., Blumenfeld P. C., Paris A. H. (2004). School engagement: Potential of the concept, state of the evidence. Review of Educational Research.

[B17-behavsci-16-00488] Goldman S. R., Taylor J., Carreon A., Smith S. J. (2024). Using AI to support special education teacher workload. Journal of Special Education Technology.

[B18-behavsci-16-00488] Hayes A. F. (2018). Introduction to mediation, moderation, and conditional process analysis: A regression-based approach.

[B19-behavsci-16-00488] Hou C., Zhu G., Sudarshan V. (2025). The role of critical thinking on undergraduates’ reliance behaviours on generative AI in problem-solving. British Journal of Educational Technology.

[B20-behavsci-16-00488] Kong L. (2023). A study on the current situation and influencing factors of informatization teaching ability of special education pre-service teachers. Master’s thesis.

[B21-behavsci-16-00488] Larson J. R., Christensen C. (1993). Groups as problem-solving units: Toward a new meaning of social cognition. British Journal of Social Psychology.

[B22-behavsci-16-00488] Li Y., Xu J., Du M. R. (2025). Typical characteristics and group classification of college students using GenAI. Modern Educational Technology.

[B23-behavsci-16-00488] Lim J., Lee U., Koh J., Jeong Y., Lee Y., Byun G., Jung H., Jang Y., Lee S., Moon J. (2025). Development and implementation of a generative artificial intelligence-enhanced simulation to enhance problem-solving skills for teacher trainees. Computers & Education.

[B24-behavsci-16-00488] Liu B., Xu L., Luo X., Lu S. (2023). Research on the relationship between teacher support and learning engagement in chinese high school information technology courses: Mediation effect analysis based on computer self-efficacy. International Journal of Information and Communication Technology Education (IJICTE).

[B25-behavsci-16-00488] Liu Y., Zhang Z., Wu Y. (2025). What drives Chinese university students’ long-term use of GenAI? Evidence from the heuristic-systematic model. Education and Information Technologies.

[B26-behavsci-16-00488] Lu G., Xie K., Liu Q. (2022). What influences student situational engagement in smart classrooms: Perception of the learning environment and students’ motivation. British Journal of Educational Technology.

[B27-behavsci-16-00488] Mariyono D., Alif Hidayatullah A. N. (2025). Navigating the moral maze: Ethical challenges and opportunities of generative chatbots in global higher education. Applied Computational Intelligence and Soft Computing.

[B28-behavsci-16-00488] Martínez C. M., Roger-Monzo V., Castelló-Sirvent F. (2025). Generative AI and critical thinking in online higher education: Challenges and opportunities [IA generativa y pensamiento crítico en la educación universitaria a distancia: Desafíos y oportunidades]. Revista Iberoamericana de Educación a Distancia.

[B29-behavsci-16-00488] Moșoi A. A., Maican C. I., Cazan A.-M., Sumedrea S. (2025). Do students need to think hard? The interplay of AI and cognitive abilities in solving problems. Education and Information Technologies.

[B30-behavsci-16-00488] Pellas N. (2025). The role of students’ higher-order thinking skills in the relationship between academic achievements and machine learning using generative AI chatbots. Research and Practice in Technology Enhanced Learning.

[B31-behavsci-16-00488] Pintrich P. R. (1991). A manual for the use of the motivated strategies for learning questionnaire (MSLQ).

[B32-behavsci-16-00488] Rosenbaum M. (1980). A schedule for assessing self-control behaviors: Preliminary findings. Behavior Therapy.

[B33-behavsci-16-00488] Santiago C. M. (2025). ‘Generative AI made me do this’ exploring the potential of ChatGPT-assisted collaborative action research in science higher education: A case in the Philippines. Educational Action Research.

[B34-behavsci-16-00488] Sheng J. (2024). A study on the influence of metacognitive monitoring on learning engagement and intervention among undergraduates. Master’s thesis.

[B35-behavsci-16-00488] Su F. G. (2025). Risks and governance of generative artificial intelligence promoting higher education development from the perspective of complex systems. Research in Higher Education of Engineering.

[B36-behavsci-16-00488] Sun Y., Sheng D., Zhou Z., Wu Y. (2024). AI hallucination: Towards a comprehensive classification of distorted information in artificial intelligence-generated content. Humanities & Social Sciences Communications.

[B37-behavsci-16-00488] Voultsiou E., Moussiades L. (2025). A systematic review of AI, VR, and LLM applications in special education: Opportunities, challenges, and future directions. Education and Information Technologies.

[B38-behavsci-16-00488] Wan K., Rao A. J., Xu R. M. (2021). What factors affect learners’ online learning engagement?—Also on the development of online learning in the intelligent era. Education Academic Monthly.

[B39-behavsci-16-00488] Wang X., Zainuddin Z., Hai Leng C. (2025). Generative artificial intelligence in pedagogical practices: A systematic review of empirical studies (2022–2024). Cogent Education.

[B40-behavsci-16-00488] Wang Y. Y., Chuang Y. W. (2024). Artificial intelligence self-efficacy: Scale development and validation. Education and Information Technologies.

[B41-behavsci-16-00488] Wei S. H., Li Q. (2025). Analysis of digital policies for special education in the United States, Finland, Singapore, and South Korea. Chinese Journal of Special Education.

[B42-behavsci-16-00488] Wen Z., Ye B. (2014). Testing methods for moderated mediation models: Competition or substitution?. Acta Psychologica Sinica.

[B43-behavsci-16-00488] Wu F., Dang Y., Li M. (2025). A systematic review of responses, attitudes, and utilization behaviors on generative AI for teaching and learning in higher education. Behavioral Sciences.

[B44-behavsci-16-00488] Yuan P. L., Chen Y. Z., Wang X. Z., Song H. (2025). Expectation or threat: An empirical analysis of pre-service teachers’ AI awareness types and their TPACK level differences. Teacher Education Research.

[B45-behavsci-16-00488] Zhang M. K., Huang R. X., Wu X. L. (2021). An empirical study on the relationship between college students’ learning engagement and learning self-efficacy. Education Academic Monthly.

[B46-behavsci-16-00488] Zhang Y., Lai C., Gu M. M. Y. (2025). Becoming a teacher in the era of AI: A multiple-case study of teacher trainees’ investment in AI-facilitated learning-to-teach practices. System.

[B47-behavsci-16-00488] Zhou H., Long L. (2004). Statistical tests and control methods for common method biases. Advances in Psychological Science.

[B48-behavsci-16-00488] Zhou X., Lou Z. (2021). A study on the relationship between online learning self-efficacy and deep learning among university students. Modern Education Management.

[B49-behavsci-16-00488] Zhou X., Teng D., Al-Samarraie H. (2024). The mediating role of generative AI self-regulation on students’ critical thinking and problem-solving. Education Sciences.

